# Neuroprotective Effects of Ginsenoside Rb1 on High Glucose-Induced Neurotoxicity in Primary Cultured Rat Hippocampal Neurons 

**DOI:** 10.1371/journal.pone.0079399

**Published:** 2013-11-01

**Authors:** Di Liu, Hong Zhang, Wenjuan Gu, Yuqin Liu, Mengren Zhang

**Affiliations:** 1 Department of Traditional Chinese Medicine, Peking Union Medical College Hospital, Peking Union Medical College and Chinese Academy of Medical Sciences, Beijing, China; 2 Department of cell resource center, Institute of Basic Medical Science, Peking Union Medical College and Chinese Academy of Medical Sciences, Beijing, China; Macau University of Science and Technology, Macau

## Abstract

Ginsenoside Rb1 is one of the main active principles in traditional herb ginseng and has been reported to have a wide variety of neuroprotective effects. Endoplasmic reticulum (ER) stress has been implicated in neurodegenerative diseases, so the present study aimed to observe the effects of ginsenoside Rb1 on ER stress signaling pathways in high glucose-treated hippocampal neurons. The results from MTT, TUNEL labeling and Annexin V-FITC/PI/Hoechst assays showed that incubating neurons with 50 mM high glucose for 72h decreased cell viability and increased the number of apoptotic cells whereas treating neurons with 1 μM Rb1 for 72h protected the neurons against high glucose-induced cell damage. Further molecular mechanism study demonstrated that Rb1 suppressed the activation of ER stress-associated proteins including protein kinase RNA (PKR)-like ER kinase (PERK) and C/EBP homology protein (CHOP) and downregulation of Bcl-2 induced by high glucose. Moreover, Rb1 inhibited both the elevation of intracellular reactive oxygen species (ROS) and the disruption of mitochondrial membrane potential induced by high glucose. In addition, the high glucose-induced cell apoptosis, activation of ER stress, ROS accumulation and mitochondrial dysfunction can also be attenuated by the inhibitor of ER stress 4-phenylbutyric acid (4-PBA) and anti-oxidant N-acetylcysteine(NAC). In conclusion, these results suggest that Rb1 may protect neurons against high glucose-induced cell injury through inhibiting CHOP signaling pathway as well as oxidative stress and mitochondrial dysfunction.

## Introduction

Substantial evidence from epidemiological studies suggests that diabetes is an independent risk factor for cognitive dysfunction[1]. Compared to people without diabetes, people with diabetes have a greater rate of decline in cognitive function and a greater risk of cognitive decline[2]. The importance of chronic hyperglycaemia in pathogenesis of diabetic cognitive impairment has been well established, which can not only increase polyol pathway flux and oxidative stress[3], but also enhance formation of advanced glycation end-products (AGEs) [4]and disturbances of neuronal Ca^2+^ homeostasis[5]. Both clinical studies and animal experiments revealed that diabetes-induced impairments in hippocampus are closely associated with cognitive deficits[6–8]. It also has been confirmed that the degeneration and apoptosis of hippocampal neurons played a key role in the learning and memory deficits in diabetic animals[9–12]. 

Endoplasmic reticulum (ER) is the consequence of a mismatch between the load of un-folded and misfolded proteins in the ER and the capacity of the cellular machinery that copes with that load. Under stress conditions where the ER protein folding machinery is impaired, unfolded or misfolded proteins accumulate in the ER, and this warning signal triggers the unfolded protein response (UPR) to restore ER functions via activation of three ER transmembrane receptors namely protein kinase RNA (PKR)-like ER kinase ( PERK) , inositol requiring enzyme-1 (IRE1) and activating transcription factor (ATF6)[13]. If the stress is severe or prolonged, UPR can eventually result in the activation of ER-associated apoptotic pathways involving transcriptional induction of C/EBP homology protein (CHOP), activation of the caspase-12 and c-Jun N-terminal kinase[13]. Probably the most significant ER stress-induced apoptotic pathway is mediated through CHOP, which can be induced by PERK translation and result in the downregulation of Bcl2 expression to promote cell apoptosis[14]. Recent studies also suggest that oxidative stress and mitochondrial dysfunction may provide significant contributing factors to ER stress-induced apoptosis and there were pathways connecting UPR signaling, mitochondrial dysfunction and oxidative stress during the ER stress[15–17]. Increasing evidence suggests that ER stress and cell death mechanisms play important roles in the etiology of numerous disease states, including metabolic disease (diabetes, obesity, atherosclerosis)[18] and neurodegenerative disease (Alzheimer's and Parkinson's disease)[19]. Data suggestive of a connection between ER stress and cognitive impairment have been reported in a diet-induced obese mouse model and a murine model of type 2 diabetes[20,21]. Moreover, CHOP-dependent ER stress-mediated apoptosis is implicated in hyperglycemia-induced hippocampal synapses and neurons impairment and promote the diabetic cognitive impairment[22]. Thus, therapeutic interventions targeting ER stress are receiving major attention as promising strategies in the treatment of diabetic cognitive impairment.

Ginseng, the root of Panax ginseng C.A. Meyer (Araliaceae), has been extensively used in traditional oriental medicine for over 2000 years. Ginsenoside Rb1 is generally recognized as one of the principle bioactive ingredients in ginseng and has received a great deal of attention owing to its biological properties, especially the various neuroprotective effects. A wealth of studies indicated that ginsenoside Rb1 could promote neurite out-growth and prevent MPP^+^-induced apoptosis in PC12 cells[23], enhance neurotransmitter release[24], protect neurons from ischemia[25], inhibit autophagy in glutamate-injured cortical neurons[26], and increase synapse number and the density of synaptophysin[27], which is the morphological basis for explaining Rb1 induced facilitation of learning and memory. Recent researches have also revealed that Rb1 can up-regulate cell genesis in hippocampal subregions to enhance the spatial learning and memory of rats[28].

With this background, the present study was undertaken to investigate whether Ginsenoside Rb1 could ameliorate high glucose-induced neurotoxicity, and effects of Rb1 on endoplasmic reticulum stress and the crosstalk among ER stress, oxidative stress and mitochondrial dysfunction in high glucose-treated primary cultured rat hippocampal neurons.

## Materials and Methods

### Materials

**Figure 1:**
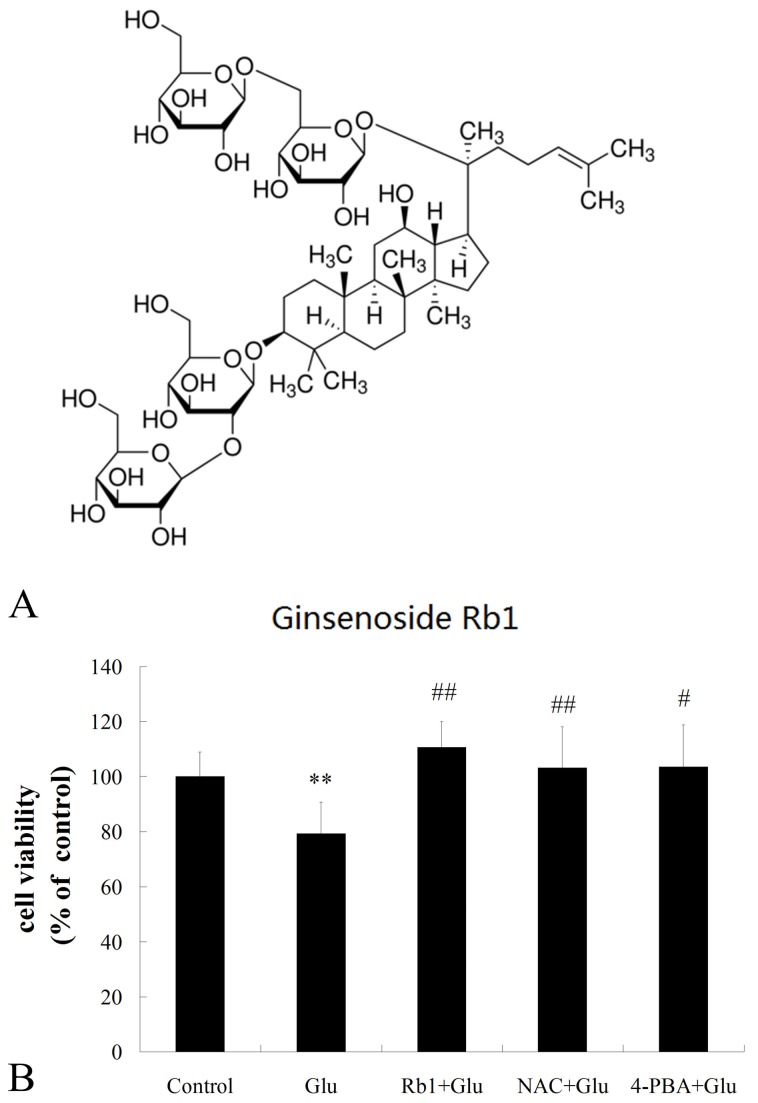
Neuroprotective effects of ginsenoside Rb1 on cytotoxicity induced by high glucose. (A) Chemical structure of ginsenoside Rb1. (B) Primary hippocampal neurons were treated with ginsenoside Rb1(1μM) in the presence of 50 mM high glucose for 72h. Cell viability was measured using the MTT assay. The results represent the mean ± S.D of at least three independent experiments, and are expressed as percentage of control; **P<0.01, as compared to the control group; ^#^ P<0.05, as compared to the high glucose group ; ^##^P<0.01, as compared to the high glucose group.

Ginsenoside-Rb1 standard was obtained from National Institute for the Control of Pharmaceutical and Biological Produces (Beijing, China). The structure of ginsenoside Rb1 (2-O-β-Glucopyranosyl-(3β, 12β)-20-[(6-O-β -D-glucopyranosyl-β-D-glucopyranosyl) oxy]-12-hydroxydammar-24-en-3-yl β -D-glucopyranoside) is illustrated in Figure 1A. All cell culture reagents were from Peking union cell center (Beijing, China). In Situ Cell Death Detection Kit was purchased from Roche Diagnostics GmbH (Penzberg, Germany). Annexin V-FITC/PI detection kit, Hoechst 33258, fluorescent probe 2′, 7′-dichlorofluorescin diacetate (DCFH-DA) and 4-phenylbutyric acid (4-PBA) were obtained from Sigma-Aldrich (St. Loius, MO, USA). N-acetylcysteine(NAC) was purchased from TCI chemicals (Shanghai, China). Mitochondrial membrane potential assay kit with 5,5′,6,6′-tetrachloro- 1,1′ ,3,3′-tetraethyl benzimidazolcarbocyanine iodide (JC-1) was obtained from Beyotime Institute of Biotechnology(Shanghai, China). Anti-p-PERK, anti-PERK, anti-CHOP and anti-Bcl-2 antibodies were from Santa Cruz Biotechnology (Santa Cruz, CA, USA). 

### Cells Culture and Treatments

All procedures were approved by the Peking Union Medical College Hospital and Chinese Academy of Medical Sciences ethical committee (D-001) and were performed according to the regulations of laboratory animal management promulgated by the Ministry of Science and Technology of the People’s Republic of China [1988] No. 134, which conforms to the internationally recognized NIH guidance for care and use of laboratory animals. Animals were sacrificed by cervical dislocation, and all efforts were made to minimize suffering.

Primary hippocampal neuronal cultures were prepared as described previously [29,30] with some modifications. Newborn Sprague-Dawley (SD) rats, less than 24 h old, were purchased from the Peking University Health Science Center (Beijing, China). Hippocampi were dissected from the brain on ice and minced in sterile ice-cold D-Hanks' with the blood vessels and meninges carefully removed. The tissues were digested with 0.25% trypsin for 15 min at 37 °C and then the digestion procedure was stopped by adding 5ml fetal bovine serum (FBS). Cell suspension was passed through a 200 mesh cell strainer and separated by density gradient centrifugation at for 20 min at 2,000 r.p.m. Then the cell suspension containing the desired cell fractions was centrifuged within D-Hanks' for 5 min at 1,500 r.p.m and resuspended in 15ml-20ml DMEM medium. The cells were then plated on poly-D-lysine (Sigma, USA) coated-glass coverslips, 96-well plates or 24-well plates at a density of 5×10^5^cell/ml after determining the cell density using a hemacytometer and maintained at 37 °C in a humidified 5% CO_2_ incubator(Forma, USA). Neurons were cultured in DMEM medium and supplemented with 10% FBS, 20mmol/L sodium pyruvate, 1 mmol/L glutamine, 100U/ml Penicillin, 100μg/ml streptomycin, 10ng/ml nerve growth factor (NGF)(Millpore, USA). The culture neurons were used for in vitro studies at day 6 and the percentage of neurons is approximately 85% using NSE immunostaining (Figure S1). Ginsenoside Rb1 Standard(1μM), high glucose(final concentration was 50 mM), 4-PBA(1mM), NAC(10 mM)were added to neurons for indicated time described elsewhere. The concentration of glucose is 25 mM in the control group. 

### Cell viability assays

After exposure to 50 mM high glucose and the indicated drug for 72h, the viability of cells was determined using the MTT assay system (Peking union cell resource center, China). In brief, the cells were plated on 96-well culture plates at a density of 5×10^5^ cell/ml. After the specific timeframe, 100μl 5mg/ml MTT reagent 1:10 was added to each well and then incubated for 4h at 37°C. After the incubation, the medium was removed and then the precipitated dye was dissolved in 100μl DMSO solution for another 30min at 37°C. Finally, the OD value of each well was detected at 570 nm using a MultiSkan3ELISA Reader (Thermo fisher scientific, USA). Cell viability was calculated as follows: treated group OD/24h control group OD× 100%. Data are presented as means ± S.D. of at least three independent experiments.

### Apoptosis detection using TUNEL staining

To confirm the presence of cell death by an apoptotic-like mechanism following exposure to 50 mM high glucose and the indicated drug we performed in situ labeling of TUNEL-positive nuclei using the In Situ Cell Death Detection Kit, POD (Roche,USA) according to the manufacturer's protocol. In brief, culture media in the wells was removed after treatments, and the cells were rinsed three times with PBS followed by addition of freshly prepared 4% paraformaldehyde in PBS for 20 min at room temperature and 0.1% Triton X-100 in 0.1% sodium citrate for 2 min on ice, after that the cells were incubated with 50 μl of TUNEL reaction mixture, incubated for 60 min in a humidified chamber for 60 min at 37 °C, and rinsed three times with PBS. Samples for fluorescence staining were followed by fluorescent counterstaining with DAPI while samples for DAB staining were treated with anti-fluorescein anti-body conjugated horse-radish peroxidase and developed with DAB for 2 min followed by hematoxylin staining. Negative controls were performed by substituting distilled water for TdT in the working solution. TUNEL staining was observed by inverted fluorescence microscopy (LEICA DMI4000B, Japan) and photographed using Image-Pro Plus 6.0 imaging system. For each preparation six random fields were counted and data are expressed as the ratio of apoptotic neurons to total neurons of at least three independent experiments. 

### Annexin V-FITC/ PI staining

Annexin V -FITC/ PI staining was performed with a kit from Sigma-Aldrich (St. Loius, MO, USA) following the instructions provided by the manufacturer. Briefly, cells were harvested and resuspended in binding buffer (10^6^ cells/mL) after being stained with Hoechst 33342 (Sigma, USA). Then aliquots of 10^5^ cells were mixed with 5 μL of Annexin V-FITC and 10 μL of PI. After 15 min in the dark at room temperature, the preparations were visualized with a live cell imaging system (Olympus LCS SYSTEM, Japan) and photographed using a volocity demo imaging system (PerkinElmer, USA). For each preparation, at least 6 random fields were counted. The percentage of apoptosis was determined from the number of Annexin V(+) /PI(−) cells relative to the number of total cells. The experiments were repeated three times independently.

### Western blot analysis

After treating hippocampal neurons as described above, total protein was extracted from cell lysates using RIPA buffer (50 mM Tris –HCl, pH 7.4, 150 mM NaCl, 1% sodium deoxycholate, 1 mM EDTA, 0.1% SDS, 5 μg/ml aprotinin and 5 μg/ml leupeptin). Supernatant protein concentrations were determined with Bio-Rad DC Protein Assay Kit 1, and then adjusted to the same concentration with 0.9% NaCl. A total of 40 μg of protein was resolved by 15% SDS–polyacrylamide gel electrophoresis (PAGE) and then transferred to a nitrocellulose membrane. Membranes were blocked with 5% skim milk in Tris-buffered saline with 0.1% Tween-20(TBST) for 1 h at room temperature before overnight incubation with primary antibodies specific for p-PERK (1:100), PERK (1:100), CHOP (1:200) and Bcl-2(1:200) in 5% skim milk-TBST. Membranes were then rinsed 3 times in TBST and were incubated with horseradish peroxidase-conjugated secondary IgG (1:1000) in TBST for 1.5 h, followed by a final series of rinses in TBST. Bands of protein were visualized using an enhanced chemiluminescence ECL kit (Roche, Penzberg, Germany) and were quantified by Quantity One software. Values represented the mean ± SD of triplicate determinations.

### Real-time PCR analysis

Total RNA was prepared from cells by use of the Trizol reagent according to the manufacturer's instructions. The concentration and purity of RNA were determined spectrophotometrically at 260 nm. Single-strand cDNA was prepared from 2μg of total RNA according to the protocol of the kit. Primers were designed using Primer Premier 5.0 software and their sequences were as follows: Bcl2, 5'-CTGGCATCTTCTCCTTCCAG-3', 5'-CGGTAGCGACGAGAG -AAGTC-3'; CHOP, 5'- CCTAGCTTGGCTGACTGAGG -3', 5'- CTGCTCCTTCTCCTTCATGC -3'; GAPDH, 5'- TGGTATCGTGG -AAGGACTCA -3', 5'- CCAGATGAGGCAGGGATGAT -3'. In brief, the PCR was carried out in a 20 μl final volume containing the following: 10 μl of 2× SYBR Green I master mix, 1 μl of 10μM each primer, 2 μl of 1μg/μl cDNA template, and 6 μl of 0.1% diethylpyrocarbonate-treated water. The PCR conditions were as follows: initial denaturation at 95°C for 120 s, followed by 40 cycles of denaturation at 95°C for 20 s, annealing at 60°C for 20 s, and extension at 72°C for 30 s. a melting curve was obtained after amplification by holding the temperature at 65°C for 20 s followed by a gradual increase in temperature to 95°C at a rate of 0.5°C s^− 1^. GAPDH mRNA level was used as an internal quantitative control, and the level of each target gene transcript was normalized on the basis of GAPDH mRNA content. Data are presented as means ± S.D. of at least three independent experiments.

### Intracellular ROS measurement

Intracellular reactive oxygen species were monitored using the fluorescent probe DCFH-DA, which can be oxidized to the highly fluorescent compound dichlorofluorscein (DCF). Cells were incubated with 10μM DCFH-DA at 37 °C for 30 min and then were washed twice with ice-cold PBS. Cellular fluorescence intensity was quantified using a live cell imaging system (Olympus LCS SYSTEM, Japan) with excitation at 485 nm and emission at 530 nm. The experiments were repeated three times independently.

### Mitochondrial membrane potential (Δψm) measurement

The fluorescent, lipophilic and cationic probe JC-1 (Beyotime, China) was employed to measure the mitochondrial membrane potential (Δψm) of hippocampus neurons according to the manufacturer's directions. Briefly, after indicated treatments, cells were cultured in 8-well plates(Thermo Fisher Scientific, USA ) and incubated with JC-1 staining solution (5 μg/mL) for 20 min at 37 °C. Cells were then rinsed twice with JC-1 staining buffer and fluorescence intensity of both mitochondrial JC-1 monomers ( λex 514 nm, λem 529 nm) and aggregates (λex 585 nm, λem 590 nm) were detected using a live cell imaging system (Olympus LCS SYSTEM, Japan). The Δψm of neurons in each treatment group were calculated as the fluorescence ratio of red (i.e.aggregates) to green (i.e. monomers). The experiments were repeated three times independently.

### Statistical analysis

All data were expressed as mean ± S.D. Differences between groups were examined for statistical significance using a one-way ANOVA with Dunnett's test as a post hoc analysis. P<0.05 was considered significantly different.

## Results

### Neuroprotective effects of ginsenoside Rb1 on cytotoxicity induced by high glucose

To investigate the possible neuroprotective effects of ginsenoside Rb1 on high glucose-induced neuronal damage, hippocampal neurons were treated with ginsenoside Rb1 (1μM) plus 50 mM high glucose for 72h. We had observed that 72 h incubation of 1μM Rb1 only had no cytotoxicity compared with untreated control hippocampal neurons (Figure S2, S3 and S4). The results from the MTT assay (Figure 1B) showed that 50 mM high glucose induced a significant decrease in cell viability compared with control group. However, Ginsenoside Rb1 significantly improved cell viability in high glucose-treated hippocampal neurons. 

### Effects of ginsenoside Rb1 on apoptosis induced by high glucose in hippocampal neurons

**Figure 2:**
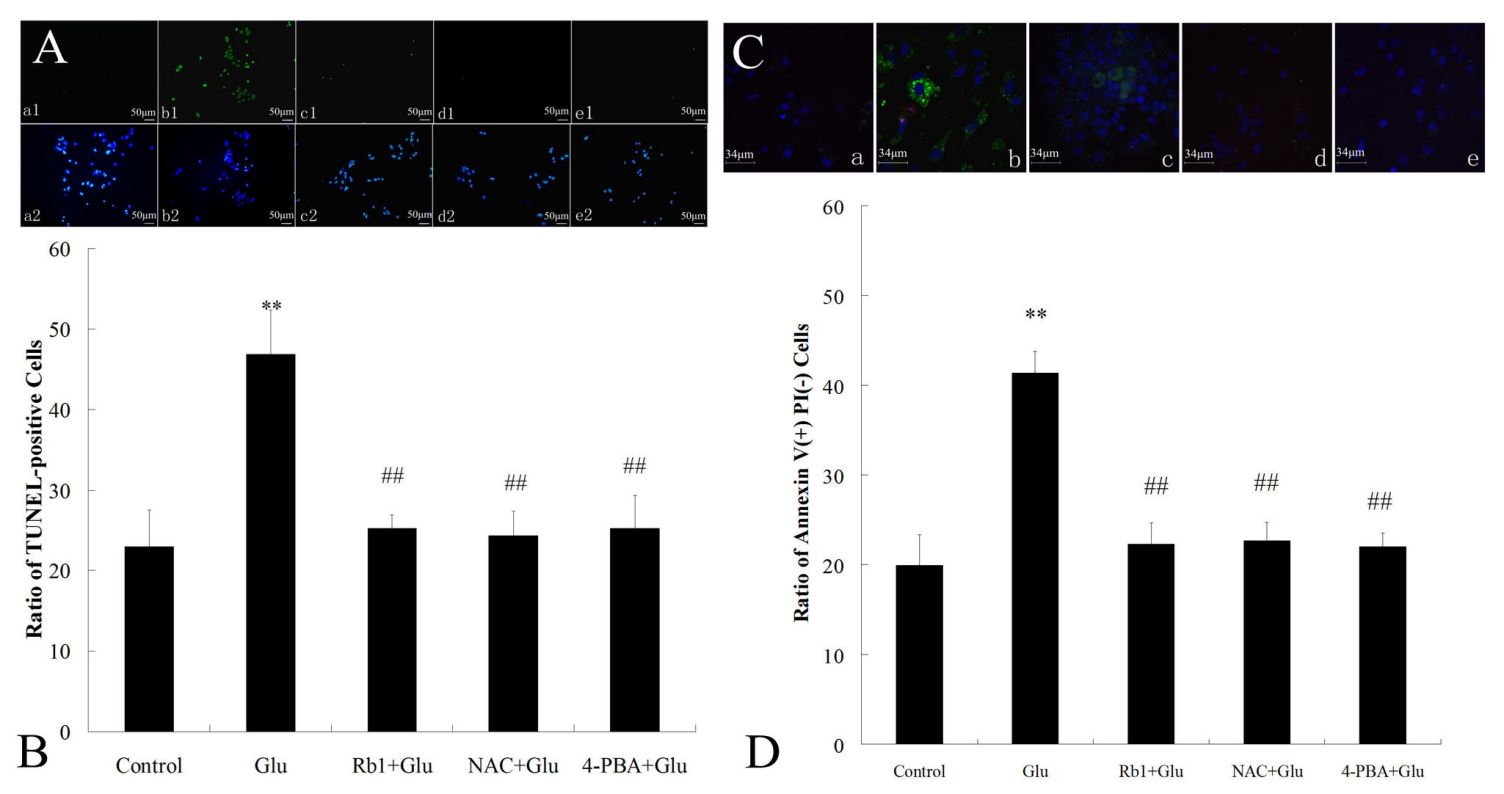
Effects of ginsenoside Rb1 on apoptosis induced by high glucose in hippocampal neurons. (A) Morphological photomicrographs of TUNEL staining. Nuclei of all cells appear blue, while TUNEL-positive (apoptotic) cells appear green. (a) control group; (b) high glucose group; (c) ginsenoside Rb1+high glucose group; (d) NAC + high glucose group;(e) 4-PBA + high glucose group. Magnification 200× ; Scale bar = 50 μm. (B) The percentage of TUNEL-positive cells in total cultured hippocampal neurons. (C) Morphological photomicrographs of Annexin V-FITC/PI assay. Nuclei of all cells appear blue, while Annexin V(+) /PI(−)(apoptotic) cells appear green. (a) control group; (b) high glucose group; (c) ginsenoside Rb1+high glucose group; (d) NAC + high glucose group;(e) 4-PBA + high glucose group. Magnification 400×; Scale bar = 34μm. (D) The percentage of Annexin V(+) /PI(−) cells in total cultured hippocampal neurons. The experiments were repeated three times independently. **P<0.01, as compared to the control group; ^##^P<0.01, as compared to the high glucose group.

We investigated the effect of ginsenoside Rb1 on high glucose-induced apoptosis in cultured hippocampal neurons using the TUNEL staining. As shown in Figure 2A, sparse numbers of TUNEL labeled cells that exhibited inter-nucleosomal DNA fragmentation were found in control conditions. In contrast, a large number of TUNEL-positive cells (green) were observed in high glucose-treated cultures. Incubation with ginsenoside Rb1(1μM) markedly reduced the number of TUNEL-labeled cells. Quantification of TUNEL staining (Figure 2B) indicated that high glucose exposure yielded 46.86%±5.51% of TUNEL-positive cells relative to total neurons, higher than the 22.98% ±4.55% observed in the control group. Moreover, when compared with high glucose exposure alone, treatment of cells with ginsenoside Rb1 significantly decreased the percentage of TUNEL-positive cells to 25.25% ± 1.71%.

The anti-apoptotic effects of ginsenoside Rb1 were further studied using an Annexin V-FITC/PI assay. The exposure of the phospholipid phosphatidylserine (PS) on the outer leaflet of the plasma membrane occurs in cells undergoing apoptosis. Because the anticoagulant protein annexin V binds with high affinity to PS, the fluorochrome-tagged annexin V is frequently used as a marker of apoptosis. In control condition, the ratio of Annexin V(+) /PI(−) cells was 19.97% ±3.35% whereas incubation with 50 mM glucose for 72h (Figure 2C) significantly increased the percentage of Annexin V(+) /PI(−) cells to 41.38% ±2.39%(Figure 2D). Treatment with ginsenoside Rb1 significantly attenuated high glucose-induced cell apoptosis and the percentage of Annexin V(+) /PI(−) cells decreased to 22.31% ±2.36%. 

### Effects of ginsenoside Rb1 on ER stress-mediated apoptotic signaling pathways in high glucose-treated hippocampal neurons

**Figure 3:**
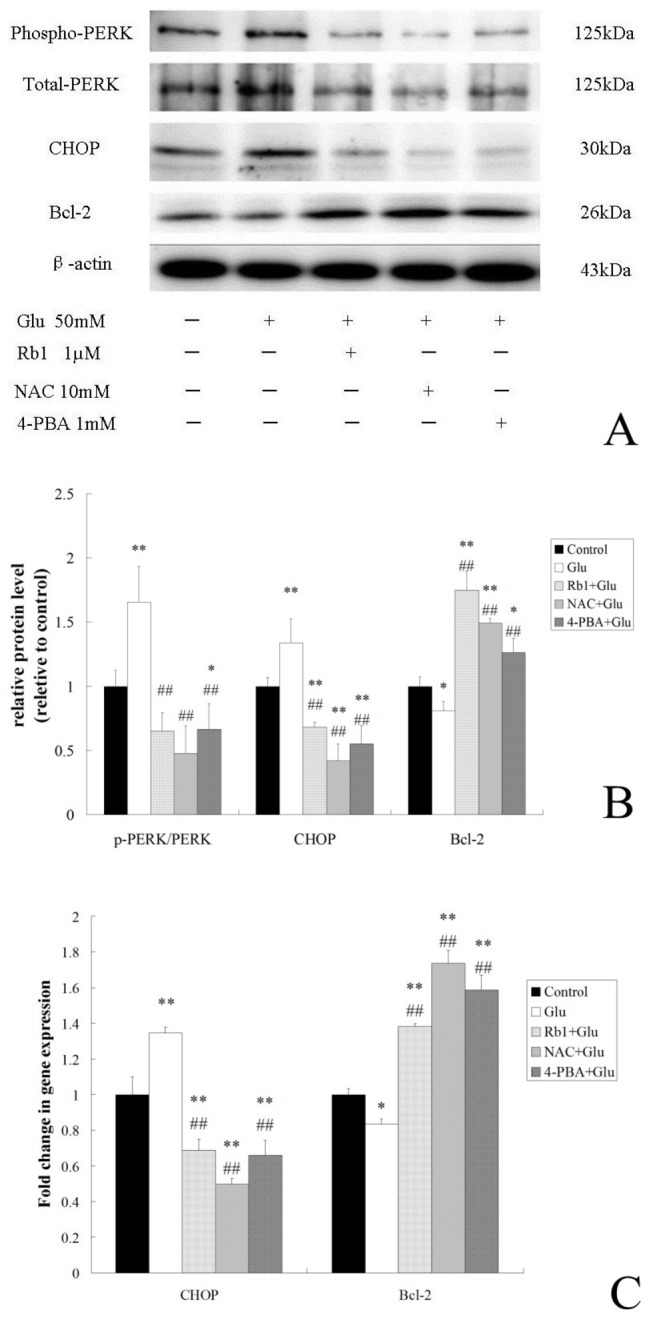
Effects of ginsenoside Rb1 on ER stress-mediated apoptotic signaling pathways in high glucose-treated hippocampal neurons. (A) Immunoreactive bands of p-PERK, PERK, CHOP, Bcl-2 and actin using specific antibody. (B) Quantitative analysis of relative protein level. Results are expressed as the means ± SD from 3 independent experiments.β-actin was used as the loading controls. (C)Histogram indicating the fold changes in mRNA levels by real-time PCR compared to the respective control after normalization to GAPDH. The results represent the mean ± S.D of three independent experiments. *P<0.05, as compared to the control group; **P<0.01, as compared to the control group; ^##^P<0.01, as compared to the high glucose group.

CHOP signaling pathway is a key modultor in ER stress-induced apoptosis and current evidence suggests that the transcription of CHOP can be induced through PERK UPR pathways and the proapoptotic activity of CHOP can be executed via dawnregulation of Bcl-2[14]. Therefore we attempted to determine whether high glucose induces activation of CHOP signaling and ginsenoside Rb1 inhibits the induction. The phosphorylation level of PERK and the expression of CHOP and Bcl-2 were assessed by Western blot analysis (Figure 3A). Compared with the control, cells exposed to high glucose for 72h showed an increased ratio of p-PERK/PERK and an increased level of CHOP expression whereas a decreased level of Bcl-2 expression(Figure 3B). In contrast, treatment with ginsenoside Rb1 effectively attenuated the high glucose-induced PERK phosphorylation, CHOP induction and dawnregulation of Bcl-2(Figure 3B). 

We further examine the effect of ginsenoside Rb1 on CHOP and Bcl-2 gene expression by real-time PCR analysis (Figure 3C). Results showed that treatment of neurons with high glucose alone resulted in significant increases in CHOP induction and decreases in Bcl-2 expression as compared to the control group. However, ginsenoside Rb1 significantly decreased the CHOP mRNA expression and increased the Bcl-2 mRNA expression. 

To understand the involvement of ER stress signaling pathway in high glucose-induced cell death, cells were treated with inhibitor of ER stress 4-PBA in the presence of high glucose for 72 h. The results from Western blot analysis and RT-PCR indicated that 4-PBA markedly inhibited PERK phosphorylation and CHOP induction while upregulated Bcl-2 expression (Figure 3). We also found that 4-PBA significantly improved cell viability and reduced the cell apoptosis in high glucose-treated hippocampal neurons (Figure 1 and 2).

### Effects of ginsenoside Rb1 on intracellular ROS accumulation induced by high glucose in hippocampal neurons

**Figure 4:**
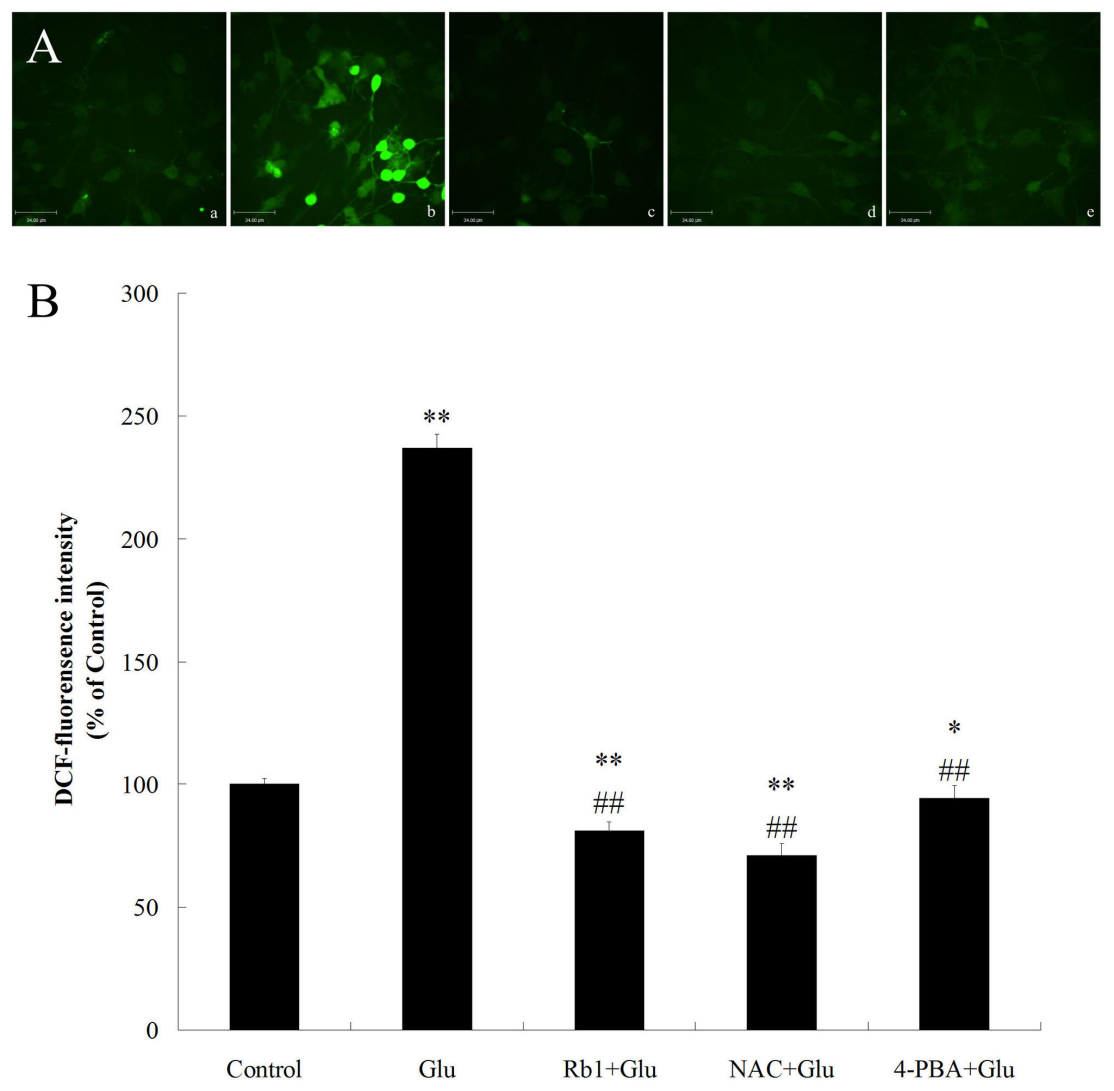
Effects of ginsenoside Rb1 on intracellular ROS accumulation induced by high glucose in hippocampal neurons. (A) Representative photographs of DCF staining in different groups. (a) control group; (b) high glucose group; (c) ginsenoside Rb1+high glucose group; (d) NAC + high glucose group;(e) 4-PBA + high glucose group. Magnification 400× ; Scale bar = 34μm. (B) DCF fluorescence intensity was quantitatively analyzed. All values are denoted as means ±S.D from six independent photographs shot in each group. Data are expressed as percentage of control group. The experiments were repeated three times independently. *P<0.05, as compared to the control group; **P<0.01, as compared to the control group; ^##^P<0.01, as compared to the high glucose group.

Recent studies have shown the involvement of ROS in ER stress-induced apoptosis[31]. Therefore, we examined the effects of ginsenoside Rb1 on the accumulation of ROS in high glucose-treated hippocampal neurons, using the DCFH-DA, which can be oxidized to the highly fluorescent compound DCF(Figure 4A). Compared with the control, incubating cells with 50mM high glucose for 24h significantly increased ROS levels (Figure 4A and 4B). However, treatment with ginsenoside Rb1 (1μM) for 24 h effectively attenuated this increase in ROS levels induced by high glucose. In addition, compared with the well-known anti-oxidant NAC, the anti-oxidative effect of ginsenoside Rb1 was similar to that of NAC (Figure 4B). These results suggest the involvement of oxidative stress in high glucose-induced cytotoxicity and the anti-oxidative activity of ginsenoside Rb1. 

To understand the roles of ROS in ER stress-mediated cell death, we also examined the effect of NAC on high glucose-induced cytotoxicity and CHOP signaling activation. Similar to ginsenoside Rb1, NAC improved cell viability, inhibited cell apoposis and also attenuated the activation of CHOP signaling in high glucose-treated hippocampal neurons (Figure 1,2 and 3). 

### Effects of ginsenoside Rb1 on mitochondrial function in high glucose-treated hippocampal neurons

**Figure 5:**
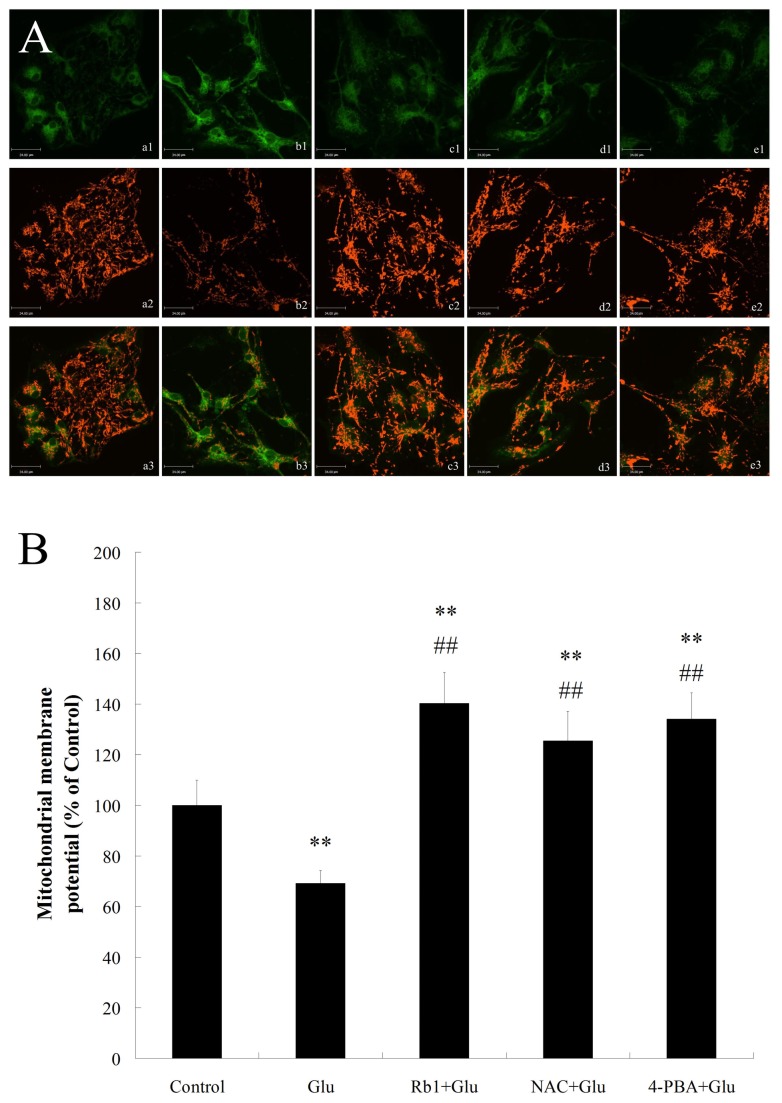
Effects of ginsenoside Rb1 on mitochondrial function in hippocampal neurons exposed to high glucose. (A) Representative photographs of JC-1 staining in different groups. (a) control group; (b) high glucose group; (c) ginsenoside Rb1+high glucose group; (d) NAC + high glucose group;(e) 4-PBA + high glucose group. Magnification 400× ; Scale bar = 34μm. (B) Quantitative analysis of the Δψm among groups. All values are denoted as means ±S.D from six independent photographs shot in each group. Data are expressed as percentage of control group. The experiments were repeated three times independently. **P<0.01, as compared to the control group; ^##^P<0.01, as compared to the high glucose group.

During ER stress, crosstalk between ER and mitochondria induces mitochondrial damage and enhances cell death[17,32]. The protective effect of ginsenoside Rb1 on mitochondrial function in high glucose-treated hippocampal neurons was measured by JC-1 staining. Control hippocampal neurons stained with JC-1 emitted mitochondrial orange-red fluorescence with a little green fluorescence (Figure 5A). However, when cells were exposed to high glucose(50mM) for 72h, the Δψm rapidly depolarized(Figure 5B), as shown by the increase in green fluorescence and the concomitant disappearance of red fluorescence(Figure 5A). Treatment with ginsenoside Rb1 (1μM) reduced the changes in Δψm(Figure 5B) as indicated by repression of green fluorescence and restoration of red fluorescence, suggesting that ginsenoside Rb1 protects hippocampal neurons by preventing high glucose-induced mitochondrial damage.

Recent studies found that prolonged ER stress can directly induce cytotoxic ROS in the cytoplasm and thus resulting in mitochondrial dysfunction to enhance cell damage[33]. To investigate whether there was link between ER stress, oxidative stress and mitochondrial dysfunction under high glucose stimulation, we also observe the effect of NAC and 4-PBA on the accumulation of ROS and mitochondrial function in high glucose-treated hippocampal neurons. Similar to the effect of ginsenoside Rb1, NAC and 4-PBA reduced high glucose-induced ROS accumulation as well as Δψm reduction in hippocampal neurons(Figure 4 and 5), suggesting the involvement of ER stress, ROS generation and mitochondrial dysfunction was implicated in high glucose-induced neurotoxicity. 

## Discussion

Ginseng has been a very important component of Chinese prescriptions for thousands of years. Ginsenoside Rb1 is one of active component in the ginseng root and its neuroprotective effects have been identified in many in vitro and in vivo studies. It has been known that Rb1 protects hippocampal neurons against either ischemia or glutamate-induced neurodegeneration[25,26]. Recent study also revealed that Rb1 regulates cell genesis in the hippocampal subregions of rats[28]. However, there is little information available in literature regarding the effects of Rb1 endoplasmic reticulum stress signaling pathways in high glucose-treated hippocampal neurons.

In this paper we have demonstrated that ginsenoside Rb1 has a neuroprotective effect on high glucose-induced neurotoxicity, which was characterized by decreasing cell viability and increasing neuronal apoptosis. The mechanisms of this neuroprotective effect may be involved in the depression of the ER stress-related apoptotic signaling pathway, alleviation of intracellular ROS generation and inhibition of mitochondrial dysfunction.

Diabetes mellitus has repeatedly been reported to be associated with cognitive deficits and an increased risk of dementia. And these deficits are paralleled by neurophysiological and structural changes in the hippocampus, the major area associated with learning and memory. Experiments in diabetic animals found that diabetes mellitus caused hippocampal neuronal apoptosis[7], reducing capacity for neurogenesis and degeneration in synaptic structures[34]. Clinical study indicated that hyperglycemia plays a critical role in hippocampus dysfunction in patients with both type 1 and type 2 diabetes mellitus[2]. Experiment on diabetic rats also has proved that blood glucose level is important for synaptic plasticity and for the ability to learn[35]. Recent study reported that long-term exposure to high glucose could induce changes in the content and distribution of some exocytotic proteins and a significant increase of apoptotic nuclei in cultured hippocampal neurons[36]. Therefore, hyperglycemia is considered the primary pathogenic factor for the development of diabetic cognitive impairment. 

In the present study, we examined primary cultured rat hippocampal neurons as a cellular model to understand the mechanisms of high glucose-induced neuronal apoptosis. A decrease in cell viability and an increase in cell apoptosis were detected in hippocampal neurons exposed to high glucose, which is consistent with previous studies where apoptosis was found in neural cells exposed to high glucose[37]. However, treatment of hippocampal neurons with 1 mM ginsenoside Rb1 significantly increased the cell viability and reduced the total number of apoptotic cells, thereby providing a neuroprotective effect.

The precise cellular mechanisms underlying high glucose-induced hippocampal neuronal damage have not been fully elucidated. Recent research suggested that endoplasmic reticulum stress was activated in the hippocampus of a murine model of type 2 diabetes[21]. One of the components of ER stress-mediated apoptotic pathway is the transcription factor CHOP, which plays an important role in diabetes, brain ischemia and neurodegenerative disease. Experiments in the diabetic rat and high glucose-treated hippocampal neurons indicated that the CHOP-dependent ER stress-mediated apoptosis maybe involved in hyperglycemia-induced hippocampal synapses and neurons impairment and promote the diabetic cognitive impairment[22]. The CHOP transcription factor features prominently in PERK pathway signaling, particularly as a marker of the UPR. It has been reported that CHOP down-regulates Bcl-2 expression and sensitizes the cells to ER stress, proving that Bcl-2 is a downstream target of CHOP signaling pathway[38]. In our study, we observed that neurons exposed to high glucose exhibited increased levels of phospho-PERK and CHOP and decreased level of Bcl-2, and ER stress inhibitor 4-PBA could reverse them. Furthermore, 4-PBA attenuated the decreasing cell viability and increasing neuronal apoptosis caused by high glucose exposure. These data indicated that activation of CHOP signaling pathway is implicated in mediating the high glucose-induced apoptosis. The results of western blot and real-time PCR analysis showed that high glucose-induced activation of CHOP signaling pathway was significantly suppressed by ginsenoside Rb1 treatment, which suggested ginsenoside Rb1 protected against high glucose-induced apoptosis via inhibition of the CHOP pathway. 

Substantial evidence has revealed that ROS production and oxidative stress are not only coincidental to ER stress, but are integral UPR components, being triggered by distinct types of ER stressors and contributing to support proapoptotic UPR signaling[15]. And studies found that another mechanism implicated in CHOP-induced apoptosis is oxidative stress[39]. Oxidation of the ER lumen is induced by the CHOP transcriptional target ER oxidase 1α (ERO1α). In the setting of diabetes, CHOP deficiency suppressed pancreatic beta cell apoptosis, and this protection was associated with decreased ERO1α, suppression of oxidative-stress markers and induction of anti oxidant genes[31]. ROS is also part of a positive feedback cycle that activates PKR and thus amplifies CHOP expression[40]. In line with this notion, we observed that intracellular ROS accumulation was significantly increased in high glucose-treated hippocampal neurons and was alleviated obviously by treatment with ginsenoside Rb1. Treatment with anti-oxidant NAC reduced high glucose-induced ROS production effectively and blocked CHOP signaling pathway as well as reduced neuronal apoptosis. Meanwhile, our results showed that 4-PBA can also attenuated high glucose-induced intracellular ROS accumulation. Collectively, these data indicated that the interaction between ER Stress and oxidative stress might play a role in high glucose-induced apoptosis and may be involved in the neuroprotective effects of ginsenoside Rb1 against high glucose. 

Recent work suggested that mitochondrial dysfunction provides a significant contributing factor to ER stress-induced neuronal apoptosis[41]. A potential mechanism linking ER stress with pro-apoptotic mitochondrial dysfunction is excess transfer of calcium from ER stores into the mitochondria, leading to Δψm[42]. It is well established that ER Stress and mitochondrial dysfunction cross-talk is associated with β-cell apoptosis in diabetes[43]. In this study, when cells were exposed to high glucose, depolarization of Δψm remarkably, indicative of mitochondrial dysfunction, was also observed and this dysfunction was alleviated significantly by treatment with ginsenoside Rb1. Prolonged ER stress can hyperoxidize the ER lumen, which may result in H_2_O_2_ leakage into the cytoplasm, and directly induce cytotoxic ROS in the cytoplasm, which could promote Ca^2+^ uptake into the mitochondrial matrix. Therefore, ROS production provides an additional mechanism by which ER stress can induce mitochondrial dysfunction[33]. Hence, we investigated whether the interactions among ER stress, oxidative stress and mitochondrial dysfunction occurred under high glucose exposure. Our findings showed that treatment of both 4-PBA and NAC markedly attenuated high glucose-induced mitochondrial depolarization, indicating that ER stress, oxidative stress and mitochondrial dysfunction interplay is involved in high glucose-induced neurotoxicity.

Recently, other ER stress pathways apart from PERK pathway have also been implicated in the pathogenesis of central nervous system complications in diabetes. One study suggested hippocampal cells adapt to type 2 diabetes-induced prolonged ER stress with partial suppression of X-box-binding protein 1 (XBP1) and glucose regulated protein-78 (GRP78/BiP) [21]. Activation of caspase-12 also been confirmed in the hippocampus of mice fed a high-cholesterol diet and ischemic brain damage associated with type 2 diabetes [44,45]. In addition, ginsenoside Rg1 was reported to upregulate GRP78/Bip expression and inhibited formaldehyde-induced CHOP increase and the decrease of pro-caspase-12 in PC12 cells[46]. Therefore, the contribution of other ER stress pathway (BiP, XBP-1 and ATF6) and extrinsic pathway medicated by UPR underlying neuroprotective effects of ginsenoside Rb1 on high glucose-induced ER stress in hippocampal neurons may warrant further investigation.

In conclusion, the present results suggest that high glucose caused neurotoxicity in primary cultured rat hippocampal neurons, through the activation of CHOP signaling pathway and the crosstalk among ER stress, oxidative stress and mitochondrial dysfunction, which could be rescued by ginsenoside Rb1 treatment. Because diabetic cognitive impairment has come into focus as an increasingly important complication of diabetes mellitus, more attention should be paid to further clarify the mechanisms of high glucose-induced brain damage and neuroprotective effects of ginsenoside Rb1, which may help to provide new insight for developing the clinical application of ginsenoside Rb1 to diabetic cognitive impairment.

## Supporting Information

Figure S1 **NSE immunostaining results.** Immunocytochemical staining with NSE for neurons, while DAPI for all cells. Magnification 200× ; Scale bar 50 μm（A and B）; Magnification 600× ; Scale bar 23 μm (C).
(TIF)

Figure S2 **Effect of exposure with 1μM Rb1 on the viability of hippocampal neurons.** Cell viability was assessed by the MTT reduction assay. The results represent the mean ± S.D of at least three independent experiments, and are expressed as percentage of control.
(TIF)

Figure S3 **Effect of exposure with 1μM Rb1 on apoptosis of hippocampal neurons.** Total cells and cells with condensed/fragmented nuclei (cells undergoing apoptosis) were counted in nine random fields in each coverslip using Hoechst staining. The results represent the mean ± S.D of at least three independent experiments, and are expressed as percentage of control.
(TIF)

Figure S4 **Neuronal morphology: (**A**) hippocampal neurons treated with normal medium (control).** (B) hippocampal neurons exposed to 1μM Rb1. Magnification 600× ; Scale bar 34 μm.
(TIF)
