# Supplementary figures and images for: Neuroprotective Effects of Ginsenoside Rb1 on High Glucose-Induced Neurotoxicity in Primary Cultured Rat Hippocampal Neurons
Source: PLoS One. 2013 Nov 1;8(11):e79399. doi: 10.1371/journal.pone.0079399 (PMC3815219; doi:10.1371/journal.pone.0079399)

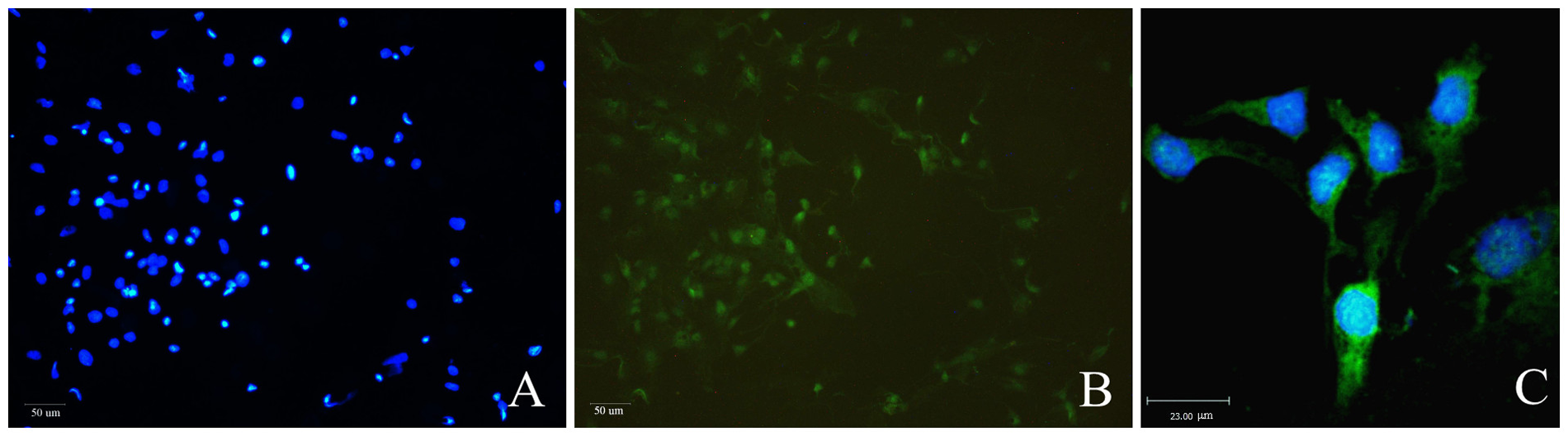

Supplement: Figure S1 — NSE immunostaining results. Immunocytochemical staining with NSE for neurons, while DAPI for all cells. Magnification 200× ; Scale bar 50 μm（A and B）; Magnification 600× ; Scale bar 23 μm (C). (TIF) [file pone.0079399.s001.tif]

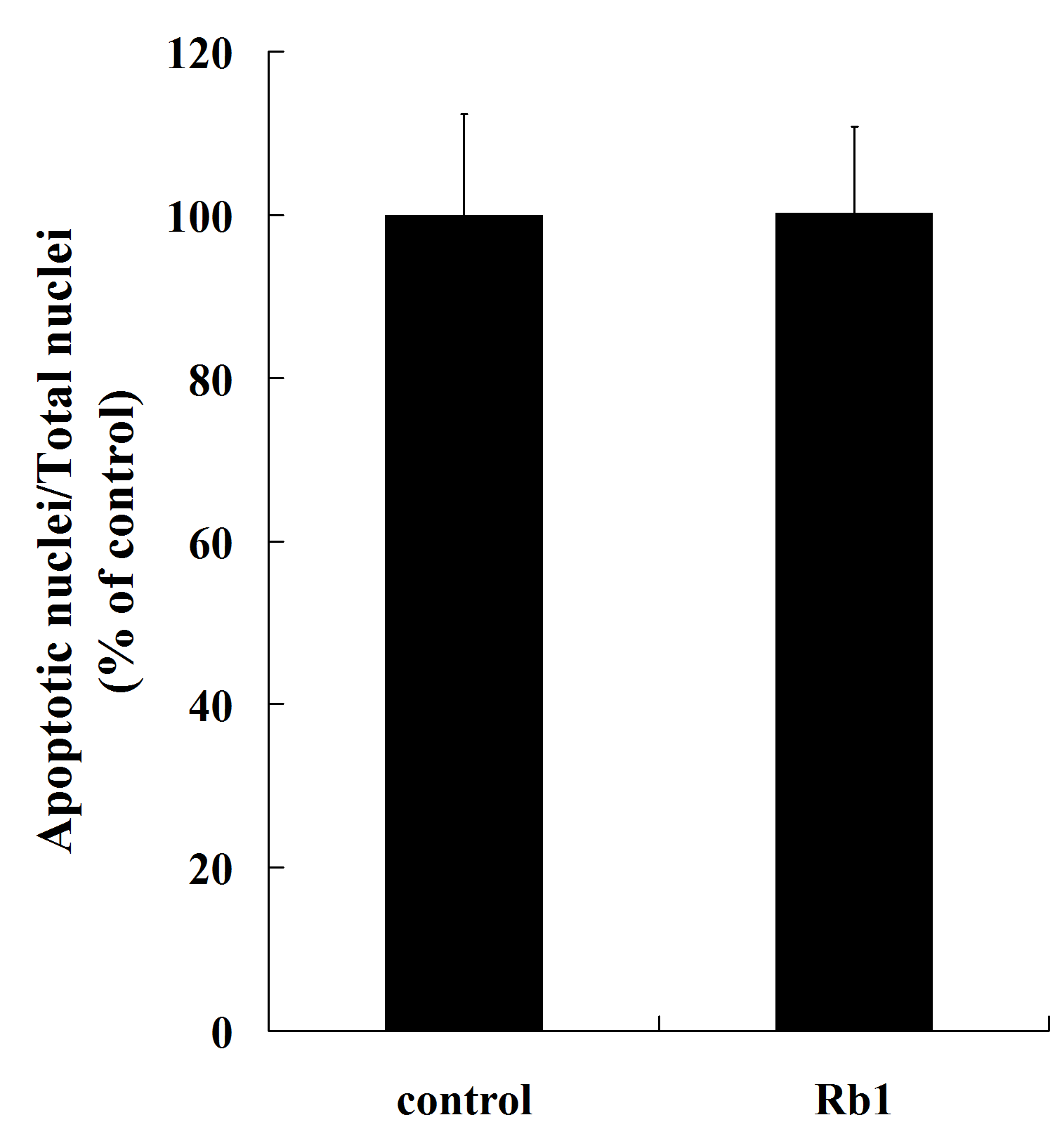

Supplement: Figure S2 — Effect of exposure with 1μM Rb1 on the viability of hippocampal neurons. Cell viability was assessed by the MTT reduction assay. The results represent the mean ± S.D of at least three independent experiments, and are expressed as percentage of control. (TIF) [file pone.0079399.s002.tif]

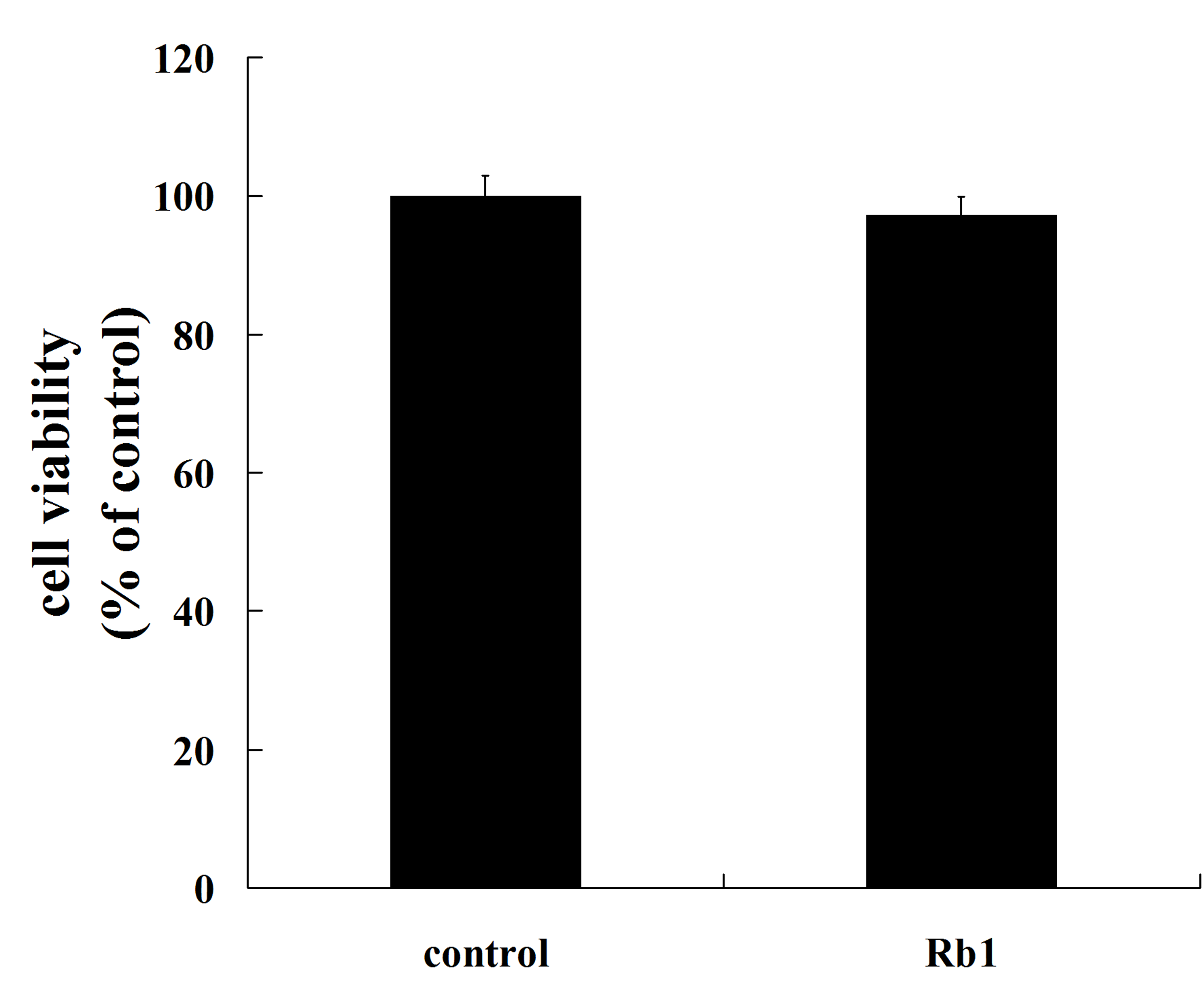

Supplement: Figure S3 — Effect of exposure with 1μM Rb1 on apoptosis of hippocampal neurons. Total cells and cells with condensed/fragmented nuclei (cells undergoing apoptosis) were counted in nine random fields in each coverslip using Hoechst staining. The results represent the mean ± S.D of at least three independent experiments, and are expressed as percentage of control. (TIF) [file pone.0079399.s003.tif]

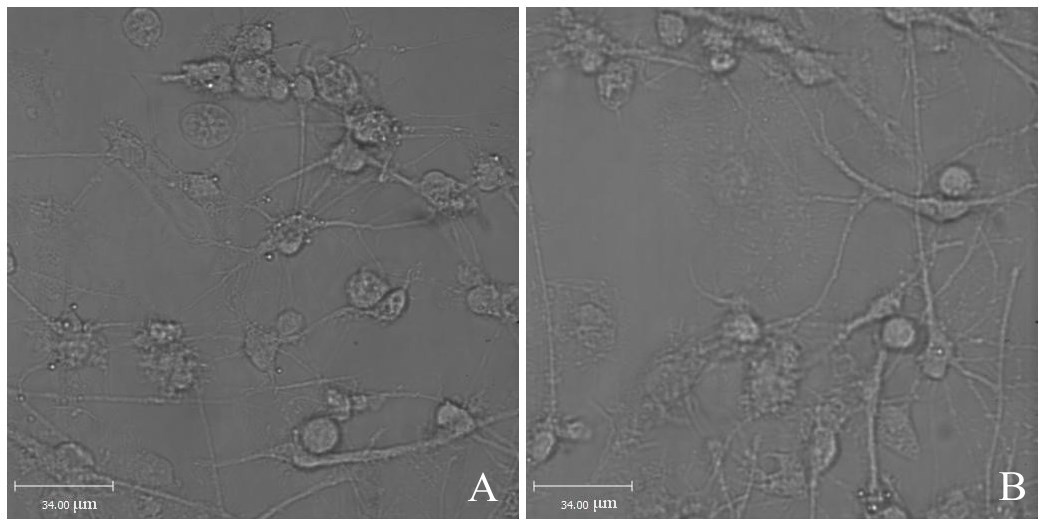

Supplement: Figure S4 — Neuronal morphology: (A) hippocampal neurons treated with normal medium (control). (B) hippocampal neurons exposed to 1μM Rb1. Magnification 600× ; Scale bar 34 μm. (TIF) [file pone.0079399.s004.tif]
